# Clinical outcomes of atrial tachyarrhythmia hospitalizations with and without prior thoracic irradiation

**DOI:** 10.3389/fonc.2026.1811487

**Published:** 2026-07-16

**Authors:** Aren Singh Saini, Sahil Ghay, Baneet Kaur, Raksha Narasimhan, Shalina Chithriki, Pooja Ghay, Kayla Samimi, Isabella Dreyfuss, Roopin Pal Singh, Brandon Mahal, Crystal Seldon Taswell

**Affiliations:** 1 Department of Radiation Oncology, Sylvester Comprehensive Cancer Center, University of Miami Miller School of Medicine, Miami, FL, United States; 2 Department of Internal Medicine, Mount Sinai Medical Center, Miami Beach, FL, United States; 3 Edward Via College of Osteopathic Medicine, Monroe, LA, United States

**Keywords:** arrhythmias, atrial fibrillation, atrial flutter, cardio-oncology, cardiovascular outcomes, in-hospital mortality, thoracic radiation

## Abstract

**Introduction:**

Thoracic radiation therapy (TRT) is commonly used for breast, lung, and lymphoid cancers. While its cardiotoxic effects, particularly coronary artery disease, are well recognized, less is known about its association with arrhythmia-related hospitalizations.

**Methods:**

A retrospective cohort study using the National Inpatient Sample (2016–2022) was conducted. Hospitalizations for atrial fibrillation or flutter were identified using ICD-10 codes. Documented prior thoracic irradiation was defined using a history of radiation therapy code in combination with thoracic malignancy codes. Propensity score matching followed by post-matching multivariable regression adjustment was used to evaluate outcomes. The primary endpoint was in-hospital mortality; secondary endpoints included length of stay (LOS) and total costs.

**Results:**

Among 3,198,304 weighted admissions, 8,570 (0.27%) had prior TRT. After matching, TRT was associated with higher odds of in-hospital mortality (adjusted odds ratio [aOR] 1.97; 95% CI 1.17–3.32; p=0.010) and longer LOS (+0.30 days; 95% CI 0.05–0.55; p=0.019) without increased costs (p=0.202). Hospitalizations with documented prior thoracic irradiation also had higher odds of palliative consultation (aOR 2.60, p<0.001) and DNR status (aOR 1.97, p<0.001), but lower odds of acute kidney injury (aOR 0.66, p<0.001).

**Discussion:**

Documented prior thoracic irradiation identified a clinically complex subgroup of atrial fibrillation or flutter hospitalizations with higher in-hospital mortality, slightly longer length of stay, and greater goals-of-care utilization.

## Introduction

Thoracic irradiation is central to the treatment of breast, lung, and lymphoid cancers. As more patients live longer after treatment, late cardiovascular toxicity has become an important long-term health concern. Radiation-associated cardiac disease includes coronary, valvular, pericardial, and myocardial injury. Radiation may also remodel atrial tissue and the cardiac conduction system. These changes can create an arrhythmogenic substrate that emerges years after therapy.

Radiation injury arises from endothelial dysfunction, microvascular rarefaction, chronic inflammation, and fibrosis. In the coronary circulation, risk rises with dose. Each 1 Gy increase in mean heart dose is associated with an approximately 7% relative increase in major coronary events, and risk persists for decades (1). Fibrotic remodeling can produce valvular stenosis or regurgitation, constrictive pericarditis, and restrictive cardiomyopathy. When injury involves the atria or the conduction system, particularly the sinoatrial node and the junction of the left atrium and the pulmonary veins, it may predispose individuals to atrial fibrillation (AF) and atrial flutter (2).

Because AF and atrial flutter are the most common sustained arrhythmias in adults, even a modest radiation-associated risk could impose a substantial inpatient burden. In the United States in 2021, there were 442,909 hospital discharges with a principal diagnosis of AF or atrial flutter (3). Both bradyarrhythmia and atrial tachyarrhythmia have been documented after thoracic irradiation. Conduction system disease appears more frequent in previously irradiated patients, consistent with fibrosis of the sinus and atrioventricular nodes (2, 4). In a retrospective lung cancer cohort treated with definitive chemoradiotherapy, a higher maximum sinoatrial node dose was independently associated with increased risk of new-onset atrial fibrillation and with worse overall survival (5). Similarly, higher left atrial and pulmonary vein doses have been correlated with greater atrial fibrillation incidence in thoracic radiotherapy cohorts (2, 6).

These observations support a dose- and location-dependent risk for atrial arrhythmias, but whether a documented history suggestive of prior thoracic irradiation is associated with short-term outcomes once atrial fibrillation or flutter leads to hospitalization remains poorly defined. Existing studies have largely focused on incidence of arrhythmia occurrence rather than prognosis during arrhythmia-related admissions (2, 7). Most are constrained by limited sample sizes, single-institution settings, and variable eras of radiation delivery, which hinder robust inference regarding in-hospital mortality, length of stay, and resource utilization.

These outcomes capture the immediate clinical burden of arrhythmia admissions and may be influenced by radiation-associated structural heart disease and oncologic complexity. Mortality risk may be compounded by the interaction of atrial arrhythmias with radiation-associated coronary artery disease, valvular dysfunction, and pulmonary comorbidity (8, 9). Length of stay and hospital costs may be affected by rhythm control and anticoagulation complexities in oncology populations, as well as differences in procedural utilization and discharge planning among previously irradiated patients. Recognizing that age, comorbidities, and cancer-related factors may confound these relationships underscores the importance of propensity score matching and multivariable adjustment in such analyses.

We therefore examined a contemporary national cohort of atrial fibrillation and atrial flutter hospitalizations to evaluate whether a documented history suggestive of prior thoracic irradiation is associated with differences in in-hospital outcomes after propensity score matching and post-matching regression adjustment. Using a large, all-payer database and International Classification of Diseases, Tenth Revision (ICD-10) coding, we evaluated whether a history of thoracic irradiation is associated with differences in in-hospital mortality, length of stay, total hospital costs, and various outcomes among hospitalizations for atrial fibrillation or flutter. This approach provides broad generalizability and complements substructure dose–response studies by addressing real-world inpatient outcomes among hospitalizations with documented prior thoracic irradiation.

## Methods

From the Agency for Healthcare Research and Quality (AHRQ), the National Inpatient Sample (NIS) is one of the largest databases available, representing data from non-government-owned short‐term, acute‐care hospitals in the United States. It is a part of the larger Healthcare Cost and Utilization Project. The NIS is released annually, comprises a stratified, nationally representative sample of approximately 20% of all hospital discharges in the U.S. Discharge weights provided by the NIS can be applied to produce estimates that reflect national trends. Information in NIS is encoded by ICD-10-CM/ICD-10-PCS coding. Information contained in the dataset includes discharge-level demographics, diagnoses, procedures, outcomes, and hospitalization costs. All data in the NIS is de-identified and publicly available; thus, this project is exempt from institutional review board approval. Because the NIS is a discharge-level database, the unit of analysis was the hospitalization.

Data from the NIS (2016–2022) were used to conduct a retrospective cohort study examining inpatient hospitalizations over this period. The STATA18 application was used for all data analysis. Adult hospitalizations among individuals aged 18 years or older were identified. Using ICD-10-CM codes beginning with I48, hospitalizations with a principal diagnosis of atrial fibrillation or atrial flutter were identified (**Supplementary 1**). Documented prior thoracic irradiation was identified using ICD-10-CM code Z92.3, history of radiation therapy, in combination with ICD-10-CM codes for thoracic malignancies (**Supplementary 1**). This definition was intended to improve specificity for documented prior thoracic irradiation in an administrative dataset; however, the NIS does not capture radiation site, dose, laterality, cardiac substructure exposure, fractionation, treatment intent, or time from radiation therapy. Baseline characteristics were collected for hospitalizations with and without documented prior thoracic irradiation, including age, gender, race, ethnicity, financial identifiers, comorbidities, and hospital characteristics such as hospital location, size, and teaching status.

To account for potential confounding, we performed 1:1 nearest-neighbor propensity score matching (PSM) without replacement, applying a caliper of 0.2. Propensity scores were calculated using multivariable logistic regression incorporating demographic, hospital, and clinical variables. These included age, race/ethnicity, gender, ZIP code–based income quartile, insurance type, Charlson comorbidity index category, and hospital characteristics (bed size, region, and teaching status). Clinical covariates included alcohol use, chronic obstructive pulmonary disease, congestive heart failure, diabetes, hepatic disease, hyperlipidemia, hypertension, peripheral vascular disease, pulmonary circulation disorders, including pulmonary hypertension, prior chemotherapy exposure, and valvular heart disease. Admissions with incomplete data for the exposure, primary outcome, or covariates used in the propensity score and outcome models were excluded using a complete-case approach. Missingness was summarized overall and stratified by documented prior thoracic irradiation status. We also compared baseline characteristics between included complete-case admissions and excluded admissions to evaluate potential selection bias. Covariate balance post-matching was assessed via standardized mean differences and variance ratios, confirming adequate matching quality (mean difference of 2.1% with all standardized mean differences <10%). After propensity score matching, outcomes were evaluated using post-matching multivariable regression adjustment with the same prespecified covariates used in the propensity score model to further reduce residual confounding. Binary outcomes were analyzed using multivariable logistic regression, while continuous outcomes were evaluated with linear regression. P-values below 0.05 were interpreted as statistically significant.

Two analyses were performed. The primary analysis compared hospitalizations with documented prior thoracic irradiation to hospitalizations without documented prior thoracic irradiation among all hospitalizations for atrial tachyarrhythmias. As an exploratory secondary analysis, we compared hospitalizations with radiation therapy only to those with chemotherapy only. This comparison was intended to provide an oncology treatment-exposed comparator, but was considered hypothesis-generating because tumor type, stage, treatment intent, time since therapy, prognosis, frailty, and baseline cardiopulmonary reserve are not fully captured in the NIS. A separate propensity score model using the same covariates was applied, with 1:1 matching performed without replacement. Binary and continuous outcomes were evaluated. Mortality was the primary outcome, and secondary outcomes included resource utilization metrics (length of stay, total costs) and clinical outcomes such as acute kidney injury, blood transfusion, cardiac arrest, DNR status, ischemic stroke, mechanical ventilation, palliative consultation, and vasopressor use. Because DNR status may represent a downstream marker of illness severity and goals-of-care decisions rather than a traditional baseline confounder, we performed a sensitivity analysis excluding hospitalizations with documented DNR status before propensity score estimation and matching. The fully adjusted mortality model was then repeated using the original covariate set. This sensitivity analysis was interpreted cautiously because exclusion of DNR admissions substantially reduced the number of mortality events.

To evaluate whether the association between a documented history of prior thoracic radiation and clinical or resource outcomes differed by tumor type, we constructed fully adjusted multivariable regression models for each prespecified endpoint: in-hospital mortality, ischemic stroke, acute kidney injury, cardiac arrest, blood transfusion, vasopressor use, mechanical ventilation, palliative care consultation, do-not-resuscitate status, total hospital costs, and length of stay. Each model included an interaction term between prior thoracic radiation and tumor type. A global Wald test was used to determine whether tumor type significantly modified the effect of prior radiation. For the mortality endpoint, tumor type was modeled as lung/airway versus all other thoracic malignancies, as lung/airway constituted the majority of cases and carries the highest baseline mortality and cardiac dose; remaining categories were pooled per HCUP cell-size standards. Tumor-type interaction analyses were considered exploratory. Tumor categories with sparse cell sizes were collapsed in accordance with HCUP reporting standards, and weighted counts were rounded to the nearest whole hospitalization. Because several tumor-specific and endpoint-specific models had sparse events, particularly for rare outcomes, nonsignificant interaction tests were not interpreted as definitive evidence of equivalent associations across tumor types. Unweighted and weighted sample sizes for each tumor category were reported in **Table 1**. Statistical significance for interaction was defined as a two-sided p < 0.05.

**Table 1:** Tumor-type distribution among atrial fibrillation or flutter hospitalizations with thoracic malignancy codes, stratified by documented prior thoracic irradiation status.

Tumor type	Unweighted total	Weighted total	Unweighted CXRT	Weighted CXRT	Unweighted no CXRT	Weighted no CXRT
Lung/Airway	9,304	46,520	1,223	6,115	8,081	40,405
Breast	2,661	13,305	263	1,315	2,398	11,990
Esophagus	685	3,425	125	625	560	2,800
Larynx	133	665	27	135	106	530
Heart/Mediastinum	126	630	18	90	108	540
Other thoracic malignancy/sparse tumor types	585	2,925	58	290	527	2,635

Distribution of tumor types among atrial fibrillation or flutter hospitalizations with thoracic malignancy codes, stratified by documented prior thoracic irradiation status. Both unweighted and weighted national estimates are shown; weighted counts were rounded to the nearest whole hospitalization. Sparse tumor categories were collapsed per HCUP cell-size standards. CXRT indicates documented prior thoracic irradiation.

To further explore effect modification, separate exploratory post-matching multivariable logistic regression models for in-hospital mortality were fitted within the propensity score–matched cohort, each including an interaction term between documented prior thoracic irradiation and one prespecified subgroup variable: sex, age category (<75 vs ≥75 years), prior chemotherapy exposure, race (White vs non-White), or primary payer status (Medicare vs non-Medicare), with adjustment for the prespecified covariates. Global Wald tests were used to assess interaction. Effect modification by tumor category (lung/airway vs other thoracic malignancy) was assessed in the analysis restricted to qualifying thoracic malignancy codes described above.

## Results

From 2016 to 2022, a total of 639,661 hospitalizations with a principal diagnosis of atrial fibrillation or atrial flutter were identified, of which 1,714 had a documented history of prior thoracic irradiation. After survey weighting, this corresponded to an estimated 3,198,304 hospitalizations nationally, including 8,570 (0.27%) with documented prior thoracic irradiation.

Among 639,661 atrial fibrillation/flutter hospitalizations in the primary cohort, 599,762 admissions were complete cases, corresponding to a weighted national estimate of 2,998,809 admissions (93.76%). A total of 39,899 admissions were excluded because of incomplete data, corresponding to a weighted estimate of 199,495 admissions (6.24%). Within the complete-case analytic cohort, 1,622 admissions had documented prior thoracic irradiation, corresponding to a weighted estimate of 8,110 admissions (0.27%), while 598,140 admissions did not have documented prior thoracic irradiation, corresponding to a weighted estimate of 2,990,699 admissions (99.73%). Missingness was primarily driven by insurance status (2.49%), race (2.36%), and ZIP code income quartile (1.51%), while missingness for mortality (0.04%), sex (0.01%), and age (<0.01%) was minimal. Missingness was generally similar between admissions with and without documented prior thoracic irradiation, including insurance status (2.57% vs 2.49%), race (1.98% vs 2.36%), and ZIP code income quartile (0.82% vs 1.51%). The prevalence of documented prior thoracic irradiation was similar between included and excluded admissions. A table detailing included and excluded admissions is provided in **Supplementary Table 2**.

Baseline characteristics of the full pre-matching cohort are shown in **Table 2**. Compared with hospitalizations without documented prior thoracic irradiation, hospitalizations with documented prior thoracic irradiation were older on average (72.5 vs 70.8 years, p<0.001) but no significant differences in gender were seen (52.9% vs 51.3%, p=0.19) (**Table 2**). Significant differences in racial distribution were observed, with a higher proportion of White admissions in the irradiated group (82.2% vs 79.9%, p<0.001) and a lower proportion of Hispanic and Asian admissions (**Table 2**). Primary payment type differed between both groups, with a greater share of admissions in the thoracic irradiation group insured by Medicare (76.0% vs 69.0%, p<0.001), and fewer with private insurance (14.2% vs 19.9%, p<0.001) (**Table 2**).

**Table 2:** Baseline demographic, clinical, and hospital characteristics of atrial fibrillation or flutter hospitalizations with and without documented prior thoracic irradiation before propensity score matching.

Demographic	AT without thoracic irradiation (n=3,189,734)	AT with thoracic irradiation (n=8,570)	P-value
Gender (%)			.19
Female	48.7%	47.1%	
Male	51.3%	52.9%	
Mean Age	70.8(SD ± 12.9)	72.5(SD ± 9.3)	<.001
Race			<.001
White	79.9%	82.2%	
Black	8.2%	9.6%	
Hispanic	5.8%	3.3%	
Asian or Pacific Islander	1.5%	1.1%	
Native American	0.4%	0.2%	
Other	2.0%	1.7%	
National Quartile for Median Household Income			.024
0-25th percentile	26.8%	25.8%	
26th to 50th percentile	26.7%	26.0%	
51st to 75th percentile	24.4%	27.0%	
76th to 100th percentile	20.5%	20.4%	
Payer			<.001
Medicare	69.0%	76.0%	
Medicaid	6.3%	6.4%	
Private insurance	19.9%	14.2%	
Self-pay/No charge/Other	4.8%	3.3%	
Hospital Region			<.001
Northeast	19.2%	18.9%	
Midwest	24.1%	28.8%	
South	41.2%	36.5%	
West	15.4%	15.8%	
Hospital Bed Size			.13
Small	21.5%	21.3%	
Medium	29.8%	27.8%	
Large	48.7%	50.9%	
Hospital Location/Teaching Status			<.001
Rural	10.2%	7.9%	
Urban/Nonteaching	21.5%	19.9%	
Urban/Teaching	68.4%	72.2%	
History of Congestive Heart Failure	44.9%	36.2%	<.001
History of Valvular Disease	21.5%	17.6%	<.001
History of Pulmonary Circulation Disorders	8.9%	10.7%	.007
History of Peripheral Vascular Disease	10.4%	12.4%	.007
History of Hypertension	80.1%	74.5%	<.001
History of Chronic Obstructive Pulmonary Disease	24.8%	45.5%	<.001
History of Diabetes	29.2%	26.3%	<.001
History of Liver Disease	3.9%	3.1%	.09
History of Hyperlipidemia	53.8%	52.4%	.24
History of Alcohol Abuse	1.0%	0.5%	.041
Prior Chemotherapy Exposure	1.1%	47.2%	<.001

Baseline demographic, clinical, and hospital characteristics of atrial fibrillation or flutter hospitalizations, compared between groups with and without documented prior thoracic irradiation before propensity score matching. Values are percentages unless otherwise indicated; age is reported as mean (SD). P-values are from chi-square tests for categorical variables and t-tests for continuous variables. Weighted national estimates are shown.

Clinically, hospitalizations with a documented history of prior radiation had lower rates of congestive heart failure (36.2% vs 44.9%, p<0.001), valvular disease (17.6% vs 21.5%, p<0.001), and hypertension (74.5% vs 80.1%, p<0.001), but markedly higher rates of chronic obstructive pulmonary disease (45.5% vs 24.8%, p<0.001) and prior chemotherapy exposure (47.2% vs 1.1%, p<0.001) (**Table 2**). Among other comorbidities, diabetes (29.2% vs 26.3%, p<.001), peripheral vascular disease (10.4% vs 12.4%, p=0.007), and alcohol use disorder (1.0% vs 0.5%, p=0.041) differed significantly between groups, whereas liver disease (3.9% vs 3.1%, p=0.09) and hyperlipidemia (53.8% vs 52.4%, p=0.24) displayed nonsignificant differences (**Table 2**).

Among atrial fibrillation or flutter hospitalizations with thoracic malignancy codes, lung/airway malignancies comprised the largest category, followed by breast and esophageal malignancies (**Table 1**). Sparse tumor categories were collapsed in accordance with HCUP reporting standards to avoid reporting small cell sizes.

### Primary analysis

**Figure 1:**
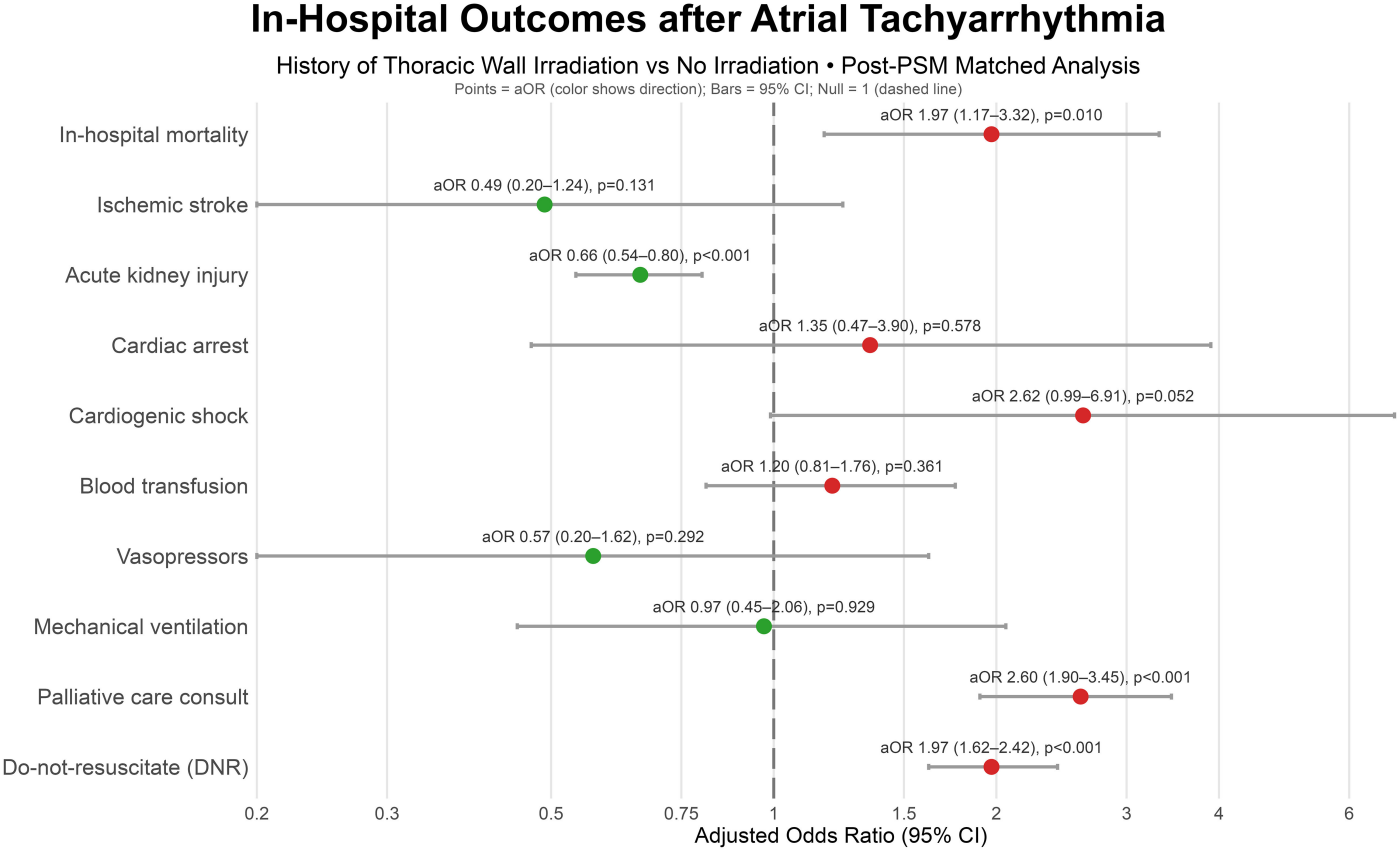
Forest plot of in-hospital outcomes after atrial tachyarrhythmia: prior thoracic radiation vs no radiation (Post-PSM analysis). Forest plot of adjusted odds ratios (binary outcomes) with 95% confidence intervals comparing in-hospital outcomes among hospitalizations with versus without documented prior thoracic irradiation, after propensity score matching with post-matching multivariable regression adjustment. The reference group is hospitalizations without documented prior thoracic irradiation; odds ratios greater than 1 indicate higher odds in the irradiated group. Length of stay and total costs are reported as adjusted mean differences and are not shown on the odds-ratio scale.

In the propensity-matched cohort, prior thoracic irradiation was associated with nearly twice the odds of in-hospital mortality (2.8% vs 1.4%; adjusted odds ratio [aOR] 1.97, 95% CI 1.17–3.32, p=0.010) and a modest increase in length of stay (+0.30 days, 95% CI 0.05–0.55, p=0.019) (**Table 3**; **Figure 1**). Total costs were not significantly different (mean difference –$2,537, p=0.202) (**Table 3**; **Figure 1**). Hospitalizations with documented prior thoracic irradiation had significantly higher odds of palliative care consultation (9.8% vs 4.2%; aOR 2.60, p<0.001) and documented DNR status (19.9% vs 11.7%; aOR 1.97, p<0.001) but lower odds of acute kidney injury (13.1% vs 18.4%; aOR 0.66, p<0.001) (**Table 3**; **Figure 1**). Rates of ischemic stroke, blood transfusion, vasopressor use, mechanical ventilation, cardiac arrest, and cardiogenic shock were similar (**Table 3**; **Figure 1**).

**Table 3:** In-hospital outcomes after propensity score matching and post-matching multivariable regression adjustment: documented prior thoracic irradiation vs no documented prior thoracic irradiation among atrial fibrillation or flutter hospitalizations.

Outcome	Absolute risk (%)	aOR/aMD	95% CI	P-value
Mortality	2.8% vs 1.4%	1.97	1.17–3.32	.010
Total Costs ($)	49,092 vs 51,978	–$2,536.56	–$6,435.00 to +$1,361.88	.202
Length of Stay (days)	4.18 vs 3.88	+0.30	+0.05 to +0.55	.019
Ischemic Stroke	0.5% vs 0.9%	0.49	0.20–1.24	.131
Acute Kidney Injury	13.1% vs 18.4%	0.66	0.54–0.80	<.001
Cardiac Arrest	0.6% vs 0.6%	1.35	0.47–3.90	.578
Cardiogenic Shock	1.0% vs 0.4%	2.62	0.99–6.91	.052
Blood Transfusion	3.8% vs 3.1%	1.20	0.81–1.76	.361
Vasopressors	0.5% vs 0.9%	0.57	0.20–1.62	.292
Mechanical Ventilation	1.0% vs 1.0%	0.97	0.45–2.06	.929
Palliative Consults	9.8% vs 4.2%	2.60	1.90–3.45	<.001
DNR Status	19.9% vs 11.7%	1.97	1.62–2.42	<.001

Absolute risks (event rate or mean) are shown for each group as documented prior thoracic irradiation vs no documented prior thoracic irradiation, alongside adjusted odds ratios (binary outcomes) or adjusted mean differences (total costs and length of stay) with 95% confidence intervals and p-values. Binary outcomes are presented as percentages; total costs (in dollars) and length of stay (in days) are presented as group means. Models were adjusted for the same covariates used in the propensity score specification. aOR, adjusted odds ratio; aMD, adjusted mean difference; DNR, do-not-resuscitate.

In a sensitivity analysis excluding hospitalizations with documented DNR status before propensity score matching, the association between documented prior thoracic irradiation and in-hospital mortality remained directionally elevated in the fully adjusted model (OR 2.66, 95% CI 0.89–7.97; p=0.080). Precision was limited by sparse events after DNR exclusion. However, tumor-specific analyses were limited by small sample sizes and sparse events for several endpoints, particularly cardiac arrest and vasopressor use.

### Subgroup analysis

In exploratory subgroup analyses of in-hospital mortality, there was no statistically significant evidence that the association between documented prior thoracic irradiation and mortality differed by sex (p for interaction=.378), age category (<75 vs ≥75 years; p=.757), prior chemotherapy exposure (p=.692), race (White vs non-White; p=.662), or primary payer status (Medicare vs non-Medicare; p=.404). In the analysis restricted to qualifying thoracic malignancies, the model recovered the expected higher mortality of lung/airway malignancy in the non-irradiated reference group (aOR 1.64, 95% CI 1.24–2.16, p<0.001), but the irradiation-by-tumor-type interaction was not significant (lung/airway vs other thoracic; p for interaction=.334). These interaction analyses were exploratory, and the absence of statistically significant interaction should be interpreted cautiously given limited mortality events within several strata.

### Secondary analysis

**Figure 2:**
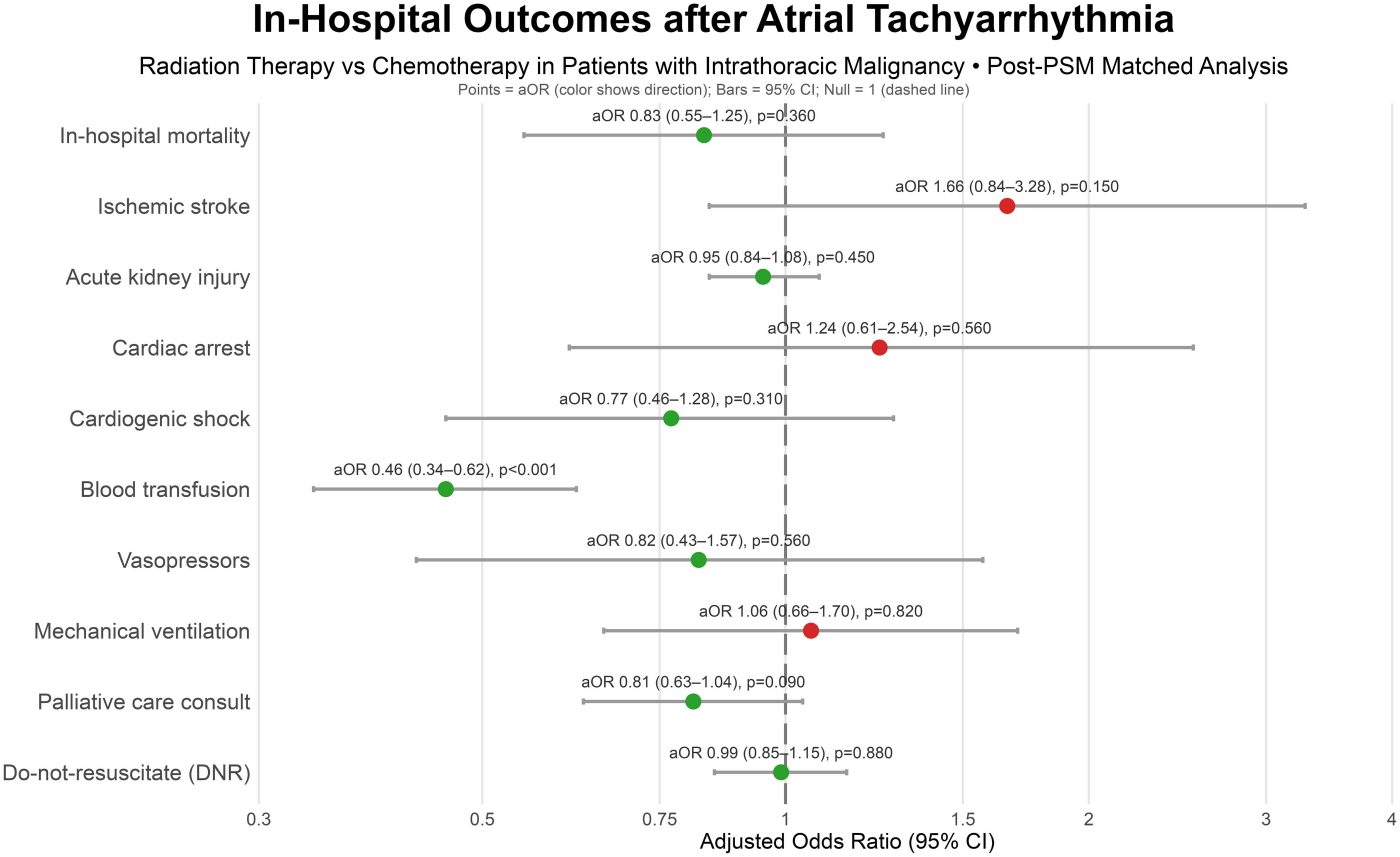
Forest plot of In-hospital outcomes after atrial tachyarrhythmia: radiation-only vs chemotherapy-only hospitalizations with intrathoracic malignancy (Post-PSM analysis). Forest plot of adjusted odds ratios (binary outcomes) with 95% confidence intervals comparing radiation-only with chemotherapy-only hospitalizations among admissions with intrathoracic malignancy codes, after propensity score matching with post-matching multivariable regression adjustment. The reference group is chemotherapy-only hospitalizations; odds ratios greater than 1 indicate higher odds in the radiation-only group. Length of stay and total costs are reported as adjusted mean differences and are not shown on the odds-ratio scale.

In an exploratory secondary analysis limited to hospitalizations with either prior thoracic radiation or chemotherapy (but not both), 1:1 propensity score matching produced 7,786 hospitalizations (3,893 per group). Radiation-only exposure was not associated with increased mortality or complications compared with chemotherapy-only exposure: in-hospital death (1.3% vs 1.5%; aOR 0.83, 95% CI 0.55–1.25), ischemic stroke, acute kidney injury, cardiogenic shock, vasopressor use, mechanical ventilation, palliative consults, and DNR status were all non-significant (**Table 4**; **Figure 2**). The only significant difference was a lower likelihood of blood transfusion in the radiation-only group (1.8% vs 3.6%; aOR 0.46, 95% CI 0.34–0.62) (**Table 4**; **Figure 2**). Total hospital costs (+ $562, 95% CI –$2,453 to $3,576) and length of stay (+ 0.03 days, –0.11 to 0.17) were similar between groups (**Table 4**; **Figure 2**).

**Table 4:** Exploratory secondary analysis of in-hospital outcomes after propensity score matching: radiation-only vs chemotherapy-only hospitalizations with intrathoracic malignancy.

Outcome	Absolute risk (%)	aOR/aMD	95% CI	P-value
Mortality	1.3% vs 1.5%	0.83	0.55–1.25	.36
Total Costs ($)	53,115 vs 52,829	+$561.70	–$2,452.50 to +$3,575.90	.72
Length of Stay (days)	3.59 vs 3.50	+0.03	–0.11 to +0.17	.68
Ischemic Stroke	0.6% vs 0.4%	1.66	0.84–3.28	.15
Acute Kidney Injury	14.3% vs 14.6%	0.95	0.84–1.08	.45
Cardiac Arrest	0.5% vs 0.4%	1.24	0.61–2.54	.56
Cardiogenic Shock	0.8% vs 1.0%	0.77	0.46–1.28	.31
Blood Transfusion	1.8% vs 3.6%	0.46	0.34–0.62	<.001
Vasopressors	0.5% vs 0.6%	0.82	0.43–1.57	.56
Mechanical Ventilation	1.0% vs 0.9%	1.06	0.66–1.70	.82
Palliative Consults	3.2% vs 3.6%	0.81	0.63–1.04	.09
DNR Status	10.4% vs 10.3%	0.99	0.85–1.15	.88

Absolute risks (event rate or mean) are shown for each group as radiation-only vs chemotherapy-only, alongside adjusted odds ratios (binary outcomes) or adjusted mean differences (total costs and length of stay) with 95% confidence intervals and p-values, comparing radiation-only with chemotherapy-only hospitalizations among admissions with intrathoracic malignancy codes. Binary outcomes are presented as percentages; total costs (in dollars) and length of stay (in days) are presented as group means. aOR, adjusted odds ratio; aMD, adjusted mean difference; DNR, do-not-resuscitate.

## Discussion

This study adds to the growing literature on radiation-associated cardiovascular disease by evaluating short-term outcomes during atrial fibrillation or atrial flutter hospitalizations among admissions with documented prior thoracic irradiation. Rather than establishing a direct causal effect of radiation exposure, our findings suggest that documented prior thoracic irradiation identifies a clinically complex cardio-oncology subgroup with higher inpatient mortality, slightly longer length of stay, and greater goals-of-care utilization (2, 10–12). These findings align with a prior analysis of acute myocardial infarction hospitalizations showing higher in-hospital mortality among hospitalizations with a documented history of prior thoracic irradiation, consistent with an association between this documented history and adverse outcomes during acute cardiovascular events (13). This study emphasizes that while radiation therapy has become a hallmark for treating breast cancer, small-cell lung cancer (SCLC), non-small cell lung cancer (NSCLC), esophageal cancers, and Hodgkin’s lymphoma, it is crucial to consider dose effects, vulnerable landmarks, co-morbidities, and interaction with other cardiotoxic therapies to optimize oncologic management and mitigate cardiac ramifications.

Prior studies have established associations between thoracic radiation exposure and later cardiovascular disease, including coronary artery disease, myocardial infarction, and atrial arrhythmias. Seminal research from Darby et al. first quantified a 7.4% increase in risk for MCE for every 1 Gy delivered to the heart with prognostic implications lasting 5–20 years post-treatment (1), which was further enhanced by evidence of TRT-related MCE independently predicting all-cause mortality (14). More recently, findings on radiation-associated atrial arrhythmias suggest that onset may occur as early as 6.7 weeks after TRT, and the occurrence of cardiotoxicity was associated with worse overall survival (HR 12.7) compared to controls (15), raising concern for increased propensity toward atrial arrhythmogenesis.

A growing body of cardio-oncology work links thoracic radiation specifically to atrial arrhythmias, and our inpatient findings extend that literature from incidence to short-term hospital outcomes. Kim et al. reported that higher sinoatrial node dose was independently associated with new-onset atrial fibrillation and worse overall survival in lung cancer (5), and Butler et al. identified left atrial and pulmonary vein dose as predictors of atrial fibrillation after thoracic radiotherapy (6). Atkins et al. showed that cardiac substructure dose was associated with both tachy- and bradyarrhythmia after lung cancer radiotherapy (16), while Apte et al. and Heckmann et al. described an increased burden of atrial fibrillation during and after thoracic irradiation (4, 7). Importantly, new-onset atrial fibrillation in oncology populations carries independent prognostic weight: a meta-analysis by Murtaza et al. found higher mortality among cancer patients who develop atrial fibrillation (17), and Zafar et al. reported higher long-term mortality among cancer survivors with prior chest radiation, in whom atrial fibrillation is a frequent presentation (18). Our analysis complements this work by showing that, once such patients are hospitalized for atrial fibrillation or flutter, a documented history of prior thoracic irradiation also identifies a subgroup with higher in-hospital mortality and greater goals-of-care utilization—a prognostic, admission-level perspective that, to our knowledge, has not previously been characterized at the national level.

While prior research contextualizes our study, this is the first to use a large nationally representative cohort of arrhythmia-related hospitalizations to evaluate inpatient outcomes among hospitalizations with a documented history of prior TRT using propensity score matching and post-matching multivariable regression adjustment. Our findings build on Guha et al., who reported increased LOS and hospital costs among hospitalizations for AF from 2003–2015 (19). Using more contemporary data (2016–2022) and including both AF and flutter, we similarly found a modestly longer LOS among hospitalizations with documented prior thoracic irradiation but no difference in total costs after matching (**Table 3**; **Figure 1**). This may reflect greater monitoring needs, discharge complexity, or differences in clinical decision-making rather than greater procedural intensity.

In the primary matched analysis, hospitalizations with documented prior thoracic irradiation had nearly twice the odds of in-hospital mortality compared to controls without prior TRT (2.8% vs 1.4%; aOR 1.97, 95% CI 1.17–3.32, p=0.010) (**Table 3**; **Figure 1**) after adjustment for age, race, insurance type, comorbidities, and hospital factors (**Table 2**). This association should be interpreted as a risk-marker finding rather than evidence of an isolated radiation-specific mortality mechanism. The higher burden of prior chemotherapy exposure, palliative care consultation, and DNR status in the irradiated group supports the likelihood that the mortality signal reflects cumulative cardio-oncology complexity in addition to any radiation-associated cardiovascular risk. Such mortality coincides with prior research from Agrawal et al., who accounted for a significant increase in incidence of atrial fibrillation (48% vs. 2.4%; p < 0.001) and high-grade AV block requiring pacemaker implantation (20% vs. 9.1%; p = 0.007) in patients with prior-TRT undergoing Transcatheter Aortic Valve Replacement (10). Together, these findings suggest that documented prior thoracic irradiation may identify a clinically higher-risk cardio-oncology subgroup during atrial tachyarrhythmia hospitalizations. Tumor-type interaction analysis (lung/airway vs other thoracic malignancies) did not detect statistically significant effect modification of the mortality association (interaction p=0.334), but this should be interpreted cautiously, as the test was underpowered for a rare endpoint and cannot establish uniformity across tumor types.

In radiation-associated cardiac disorders, traditionally, the widely regarded mechanism for such arterial and atrial injury is a sustained anti-oxidant NF-kB targeted cascade, which is triggered by imbalances in reactive oxygen species (ROS) due to radiation (20), resulting in indefinite inflammatory sequelae with upregulation of IL-6-dominant cytokine remodeling of tissues (21, 22). At the atrial level, this inflammatory and oxidative injury is thought to progress to interstitial fibrosis of the atrial myocardium and the sinoatrial and atrioventricular nodes; the resulting heterogeneity in conduction and refractoriness is hypothesized to create a re-entrant substrate that promotes atrial fibrillation and flutter, with the pulmonary vein–left atrial junction implicated as a particularly susceptible region (2, 6). Dosimetric-volume studies show that these findings hold especially true for pulmonary vein (PV) V5 dose which is significantly associated with atrial fibrillation ([sHR]: 1.04 per mL; 95% CI: 1.01–1.08; P = 0.016) and left circumflex artery (LCX) V35 dose (sHR: 1.10 per mL; 95% CI: 1.01–1.19; P = 0.028) which is significantly associated with atrial flutter (16). Because the NIS contains no radiation, biomarker, imaging, or rhythm data, these proposed pathways cannot be tested in our cohort and are presented solely as hypothesis-generating context drawn from prior mechanistic and dosimetric studies.

In addition to significant in-hospital mortality, hospitalizations with prior TRT had significantly higher rates of peripheral vascular disease (12.4% vs 10.4%, p=0.007) (**Table 2**). However, they had lower rates of congestive heart failure (36.2% vs 44.9%, p<0.001), valvular disease (17.6% vs 21.5%, p<0.001), and hypertension (74.5% vs 80.1%, p<0.001) (**Table 2**) compared to non-irradiated controls. The lower prevalence of congestive heart failure, valvular disease, and hypertension among irradiated hospitalizations despite higher mortality likely reflects residual confounding, survivor bias, or differential coding practices inherent to administrative datasets. These paradoxical findings should therefore be interpreted cautiously and not as evidence of protective remodeling. The lower observed odds of AKI among hospitalizations with documented prior thoracic irradiation were unexpected and should be interpreted cautiously. The most plausible explanation is ascertainment and competing risk: in a comfort-focused oncology population with higher palliative and DNR utilization, aggressive diagnostic workup and fluid resuscitation, the processes that generate an AKI diagnosis are pursued less often, so AKI is under-detected rather than truly less frequent. Residual confounding, differential coding, and differences in renal management may also contribute. It should not be interpreted as evidence of renal protection.

The higher odds of palliative care consultation (aOR 2.60, 95% CI 1.90–3.45; p<0.001) and documented DNR status (aOR 1.97, 95% CI 1.62–2.42; p<0.001) are important to the interpretation of mortality (**Table 3**; **Figure 1**). The higher prevalence of palliative consultation and DNR likely reflects underlying cancer severity, including malignancy burden, frailty, prognosis, and patient preferences, rather than a radiation-specific effect or arrhythmia severity alone. These variables may act as downstream markers or mediators rather than simple baseline confounders. Because these care-limitation decisions track cancer severity, they should be viewed as markers of oncologic complexity that may partially mediate the observed mortality difference, not as evidence of a radiation-specific effect or of therapeutic futility. In a sensitivity analysis excluding hospitalizations with documented DNR status before propensity score matching, the association between documented prior thoracic irradiation and mortality remained directionally elevated in the fully adjusted model, although statistical significance was attenuated and precision was limited by the small number of deaths after exclusion. Despite this loss of precision, the persistently elevated point estimate supports the primary finding that documented prior thoracic irradiation identifies a higher-risk hospitalization phenotype, while also underscoring the contribution of goals-of-care status to the observed association.

While our primary matched analysis demonstrated higher mortality and selected differences in inpatient outcomes among hospitalizations with a documented history of prior thoracic radiation compared with non-irradiated controls, additional analyses were performed to address chemotherapy as a potential confounder. In a sub-cohort limited to hospitalizations with prior thoracic radiation or chemotherapy (but not both), 1:1 propensity score matching yielded 7,786 hospitalizations (3,893 per group). Radiation-only exposure was not associated with increased mortality (OR 0.83, 95% CI 0.55–1.25, p=0.36), ischemic stroke, acute kidney injury, cardiogenic shock, or other major complications compared with chemotherapy-only exposure (**Table 4**; **Figure 2**). Resource utilization was similarly unchanged, with no difference in total hospital costs (+$562, 95% CI –$2,453 to +$3,576, p=0.72) or length of stay (+0.03 days, –0.11 to +0.17, p=0.68) (**Table 4**; **Figure 2**). The only significant finding was lower odds of blood transfusion among radiation-only hospitalizations (OR 0.46, 95% CI 0.34–0.62, p<0.001), consistent with the myelosuppression typically associated with chemotherapy (**Table 4**; **Figure 2**). These findings are supported by pre-existing literature on cardiotoxicity associated with many chemotherapy drugs (21–26), which may predispose this sub-population of hospitalizations to similar MCEs as irradiated hospitalizations.

The absence of a mortality difference between radiation-only and chemotherapy-only groups should not be interpreted as evidence that radiation is independent of chemotherapy exposure. Rather, this comparison suggests that both treatment modalities may be associated with comparable in-hospital outcomes in hospitalizations for atrial tachyarrhythmias. Because combined-modality therapy is common in clinical practice and was excluded from this comparison, potential interaction or synergistic effects cannot be excluded. Accordingly, this secondary analysis should be viewed as exploratory and hypothesis-generating rather than definitive evidence of independence. These findings should be interpreted cautiously because radiation-only and chemotherapy-only groups may differ in tumor type, stage, treatment intent, time since therapy, frailty, prognosis, and baseline cardiopulmonary reserve, which are not fully captured in the NIS.

Tumor-type interaction analyses should also be interpreted cautiously. Although we did not detect statistically significant effect modification by tumor category, the relatively small number of hospitalizations with documented prior thoracic irradiation and the rarity of several clinical endpoints limited statistical power. Sparse tumor categories were collapsed in accordance with HCUP reporting standards to avoid reporting small cell sizes. Therefore, nonsignificant interaction tests should not be interpreted as definitive evidence of uniform associations across tumor types.

This study has several limitations. Some findings could not be fully explained, such as the significantly longer length of stay among hospitalizations with documented prior thoracic irradiation compared with non-irradiated hospitalizations (+0.30 days, 95% CI 0.05–0.55, p=0.019) (**Table 3**; **Figure 1**) despite no difference in hospitalization costs (–$2,537, 95% CI –$6,435 to +$1,362, p=0.202) (**Table 3**; **Figure 1**). Similarly, the lower observed odds of diabetes (29.2% vs 26.3%, p<.001) (**Table 2**) and alcohol use disorder (1.0% vs 0.5%, p=0.041) (**Table 2**) may reflect residual confounding or coding differences. Methodologically, identification of prior thoracic radiation relied on administrative coding (ICD-10 Z92.3 in conjunction with thoracic malignancy codes) and does not provide information regarding radiation dose, cardiac substructure exposure, laterality, treatment technique, or time since therapy. Therefore, this variable should be interpreted as a documented history suggestive of prior radiation exposure rather than a quantified cardiotoxic exposure. Treatment latency could not be assessed: hospitalizations occurring soon after therapy, including those more likely to involve active malignancy, biasing mortality upward, cannot be distinguished from those among long-term survivors, in whom late radiation-associated toxicity would predominate. The exposure and cohort were defined by ICD-10 coding without internal validation. Requiring both a radiation-history code (Z92.3) and a thoracic malignancy code favors specificity but likely has low sensitivity, so truly irradiated hospitalizations missed by this definition would fall into the comparison group and bias associations toward the null, rendering our estimates conservative. Restriction to a principal diagnosis of atrial fibrillation or flutter (I48) supports cohort specificity. Sensitivity and specificity could not be quantified in the NIS. Because the NIS is a discharge-level database, repeat admissions from the same individual cannot be identified. Tumor-type interaction analyses were limited by sparse tumor-specific event counts, and nonsignificant interaction tests should not be interpreted as definitive evidence of uniform associations across tumor categories. Residual confounding by oncologic factors is a principal limitation. Active malignancy, cancer stage, metastatic burden, frailty, and performance status are not captured by the NIS, are each associated with higher mortality, and are more prevalent in the irradiated group, which by definition comprises patients with thoracic malignancy. Their omission, therefore, may bias the mortality association away from the null, so the observed estimate may overstate any radiation-specific contribution and is better read as a marker of cardio-oncology complexity. In the opposite direction, the absence of radiation dose and cardiac substructure exposure means the binary exposure pools patients with widely varying cardiotoxic dose; this misclassification would bias any true radiation-specific effect toward the null. Given these opposing biases, documented prior thoracic irradiation is best interpreted as a phenotypic risk marker rather than a precise measure of cardiotoxic dose.

In addition, any mechanistic discussion should be viewed as hypothesis-generating rather than causal, as the present dataset cannot directly evaluate biological pathways (27). Restriction to admissions with a principal diagnosis of atrial fibrillation or flutter may also introduce selection bias. Diagnostic coding position may be influenced by illness severity, oncologic complexity, or end-of-life decision-making. Hospitalizations involving advanced malignancy or care-limitation status may be more likely to have alternative principal diagnoses, potentially affecting cohort composition and comparability. Also, because combined radiation and chemotherapy exposure was not evaluated as a distinct category, potential synergistic or interaction effects between therapies cannot be excluded. The radiation-only versus chemotherapy-only comparison removes hospitalizations with a history of receiving both treatments, limiting inference regarding independence of radiation-associated risk. Finally, our analysis was limited to inpatient outcomes and did not assess post-discharge complications, late toxicities, or long-term survival.

These findings support consideration of documented prior thoracic irradiation as a marker of higher inpatient risk among atrial fibrillation or atrial flutter hospitalizations. At the bedside, this suggests that a documented radiation history, readily available from the clinical record, may serve as a simple flag identifying patients who warrant closer in-hospital observation and earlier multidisciplinary or cardio-oncology input, rather than as a basis for any specific change in arrhythmia management, which our data cannot inform. In addition to regular EKGs and advanced Holter monitoring, there is evidence that preliminary rhythm monitoring with heart rate (HR) and heart recovery rate (HRR) provides an accessible measurement that accurately characterizes autonomic dysfunction, a precursor to arrhythmias, and is linked to acute arrhythmia manifestations (28, 29). Groarke et al. found that prior TRT patients have over five times the odds of higher HRR (aOR 5.32), which was significantly associated with independently predicting an increase in all-cause mortality within 3 years (28). Furthermore, Gomez et al. suggest that transient changes in biomarkers such as BNP, which remain elevated at first follow-up (30), may provide insight into myocardial stress and warrant early onset of arrhythmogenesis and more intensive monitoring. Whether such accessible measures, HR/HRR or biomarkers such as BNP, aid risk stratification in previously irradiated patients hospitalized with atrial tachyarrhythmias is untested; we raise them as candidate tools for prospective evaluation rather than as recommendations, as our analysis cannot assess monitoring strategies.

## Conclusion

In this nationally representative cohort of atrial fibrillation and atrial flutter hospitalizations, a documented history suggestive of prior thoracic irradiation identified a clinically high-risk cardio-oncology subgroup. Hospitalizations with documented prior thoracic irradiation had higher in-hospital mortality, greater palliative care utilization, and more frequent DNR status after propensity score matching and post-matching multivariable adjustment. Although the observed mortality association likely reflects a combination of radiation-associated cardiovascular risk, oncologic complexity, and treatment burden rather than an isolated radiation-specific effect, the signal remained directionally elevated after excluding DNR admissions. These findings support closer clinical attention to patients with prior thoracic irradiation who are hospitalized with atrial tachyarrhythmias and highlight the need for future studies incorporating radiation dosimetry, cancer stage, treatment latency, and longitudinal outcomes.

## Data Availability

The original contributions presented in the study are included in the article/**Supplementary Material**. Further inquiries can be directed to the corresponding author.
