# Supplementary material for: Clinical outcomes of atrial tachyarrhythmia hospitalizations with and without prior thoracic irradiation
Source: Front Oncol. 2026 Jul 16;16:1811487. doi: 10.3389/fonc.2026.1811487 (PMC13421443; doi:10.3389/fonc.2026.1811487)
Supplement: Supplementary file 1 [file DataSheet1.pdf]

Supplementary Table 1. ICD-10-CM/PCS Codes Used to Define Exposure, Tumor Categories, Comorbidities, and Outcomes

Variable	ICD-10-CM/PCS Codes Used
Atrial fibrillation/flutter	I48x
History of radiation therapy	Z92.3
Documented prior thoracic irradiation	Z92.3 plus intrathoracic malignancy code
Prior chemotherapy exposure	Z92.21
Intrathoracic malignancy, exact codes	C33, C37, C399, C73, C761, C771, C781, C782, C7981, C7A090, C7A091, D093, D140, D141, D142, D144, D34, D491, D493, C8102, C8112, C8122, C8142, C8172, C8192, C8202, C8212, C8222, C8232, C8242, C8272, C8252, C8262, C8292, C8302, C8312, C8332, C8352, C8372, C8382, C8392, C8402, C8412, C8442, C8462, C8472, C84A2, C8492, C8512, C8522, C8582, C8592
Intrathoracic malignancy, prefix codes	C15x, C32x, C34x, C38x, C50x, C780x, C783x, D02x, D05x, D143x, D24x, D38x
Lung/Airway malignancy	C33, C399, C7A090, D142, D144, D491, C34x, C780x, C783x, D143x
Breast malignancy	C7981, D493, C50x, D05x, D24x
Esophageal malignancy	C15x
Laryngeal malignancy	C32x, D141
Heart/Mediastinum malignancy	C781, C38x
Thymus malignancy	C37, C7A091
Lymphoma	C8102, C8112, C8122, C8142, C8172, C8192, C8202, C8212, C8222, C8232, C8242, C8272, C8252, C8262, C8292, C8302, C8312, C8332, C8352, C8372, C8382, C8392, C8402, C8412, C8442, C8462, C8472, C84A2, C8492, C8512, C8522, C8582, C8592
Thyroid malignancy	C73, D093, D34
Other thoracic malignancy/sparse tumor types	C761, C771, C782, D140, D02x, D38x; sparse tumor categories collapsed for reporting
Cerebrovascular events	I60x, I61x, I63x, I65x, I66x, I67x, I68x, I69x, G45x, G46x, H34x
Intracerebral/intracranial hemorrhage	I60x, I61x, I62x, S063x, S064x, S065x, S066x
Ischemic stroke	I63x
Acute kidney injury	N17x
Palliative care consultation	Z51.5, Z71.89
Do-not-resuscitate status	Z66
Mechanical ventilation	5A1935Z, 5A1945Z, 5A1955Z
Vasopressor use	3E030XZ, 3E033XZ, 3E040XZ, 3E043XZ

Mechanical circulatory support	5A02110, 5A02210, 5A0211D, 5A0221D, 5A05121, 5A05221, 5A1522F, 5A1522G, 5A1522H
Blood transfusion	30233H0, 30233H1, 30233N0, 30233N1, 30233P0, 30233P1, 30243H0, 30243H1, 30243N0, 30243N1, 30243P0, 30243P1
Cardiogenic shock	R57.0, T81.11XA, T81.11XD, T81.11XS
Cardiac arrest	I46x, I97.120, I97.710
Cardiopulmonary resuscitation	5A12012
Congestive heart failure	I09.9, I11.0, I13.0, I13.2, I25.5, I42.0, I42.5, I42.6, I42.7, I42.8, I42.9, I43x, I50x, P29.0
Valvular disease	A52.0, I09.1, I09.8, Q23.0, Q23.1, Q23.2, Q23.3, Z95.2, Z95.3, Z95.4, I05x, I06x, I07x, I08x, I34x, I35x, I36x, I37x, I38x, I39x
Pulmonary circulation disorders / pulmonary hypertension	I28.0, I28.8, I28.9, I26x, I27x
Peripheral vascular disease	I73.1, I73.8, I73.9, I77.1, I79.0, I79.2, K55.1, K55.8, K55.9, Z95.8, Z95.9, I70x, I71x
Hypertension	I10x, I11x, I12x, I13x, I15x
Chronic obstructive pulmonary disease / chronic pulmonary disease	I27.8, I27.9, J68.4, J70.1, J70.3, J40x, J41x, J42x, J43x, J44x, J45x, J46x, J47x, J60x, J61x, J62x, J63x, J64x, J65x, J66x, J67x
Diabetes	E10.0, E10.1, E10.9, E11.0, E11.1, E11.9, E12.0, E12.1, E12.9, E13.0, E13.1, E13.9, E14.0, E14.1, E14.9, E10.2–E10.8, E11.2–E11.8, E12.2–E12.8, E13.2–E13.8, E14.2–E14.8
Liver disease	I86.4, I98.2, K71.1, K71.7, K76.0, Z94.4, B18x, I85x, K70x, K72x, K73x, K74x, K76x
Alcohol use disorder / alcohol abuse	F10x, E52x, G62.1, I42.6, K29.2, K70.0, K70.3, K70.9, Z50.2, Z71.4, Z72.1, T51x
Hyperlipidemia	E78x

Supplementary Table 2. Comparison of Included Complete-Case Admissions and Excluded Admissions With Incomplete Data

Characteristic	Excluded: incomplete data, N=39,899	Included: complete case, N=599,762
Age, years, mean (SD)	67.9 (13.3)	71.0 (12.9)
Female sex	36.2%	49.5%
Documented prior thoracic irradiation	0.2%	0.3%
In-hospital mortality	1.0%	0.9%
Length of stay, days, mean (SD)	3.3 (4.2)	3.3 (3.8)
Total charges, $, mean (SD)	48,424 (67,382)	51,473 (69,985)
Charlson comorbidity category ≥3	31.3%	33.4%
Congestive heart failure	43.7%	45.0%
Valvular disease	19.8%	21.6%
Pulmonary circulation disorders	8.1%	8.9%
Peripheral vascular disease	9.6%	10.4%
Hypertension	76.8%	80.3%
Chronic obstructive pulmonary disease	25.3%	24.9%
Diabetes	29.0%	29.3%
Liver disease	4.7%	3.8%
Alcohol use disorder	1.5%	1.0%
Hyperlipidemia	48.7%	54.2%
Prior chemotherapy exposure	1.1%	1.3%
